# Damage-induced muscle regeneration after exercise in humans: Modulatory effects of ginsenoside Rg1

**DOI:** 10.1016/j.jtcme.2026.04.002

**Published:** 2026-04-30

**Authors:** Andrew Mark Edwards, Peggy Pui Lai Or, Chien-Wen Hou, Chih-Yang Huang, Chen Zheng, Fenghua Sun, Xinmin Liu, Kunanya Masodsai, Chia-Hua Kuo

**Affiliations:** a Laboratory of Exercise Biochemistry, Education University of Hong Kong, Hong Kong Special Administrative Region; b Department of Health and Physical Education, Education University of Hong Kong, Hong Kong Special Administrative Region; c Laboratory of Exercise Biochemistry, University of Taipei, Taipei, Taiwan; d Cardiovascular and Mitochondrial Related Disease Research Center, Hualien Tzu Chi Hospital, Buddhist Tzu Chi Medical Foundation, Hualien, Taiwan; e Department of Medical Research, China Medical University Hospital, China Medical University, Taichung, Taiwan; f Department of Biotechnology, Asia University, Taichung, Taiwan; g Institute of Drug Discovery Technology, Ningbo University / Zhejiang Provincial Key Laboratory of Drug Addiction & Brain Health, Ningbo, Zhejiang, China; h School of Physical Education and Sports Science, Soochow University, Suzhou, China; i Faculty of Sport Sciences, Chulalongkorn University, Bangkok, Thailand; j Center for Molecular Medicine in Traditional Chinese Medicine, E-Da Hospital, E-Da Healthcare Group, Kaohsiung, Taiwan; k School of Chinese Medicine for Post Baccalaureate, I-Shou University, Kaohsiung, Taiwan; l Center of Excellence in Exercise Physiology for Special Populations, Chulalongkorn University, Bangkok, Thailand

## Abstract

Exercise-induced focal sarcolemmal disruption in susceptible myofibers results in bone marrow cell infiltration and reduced cellular senescence in skeletal muscle, followed by increases in muscle strength and mass. In contrast, removal of gravitational loading during spaceflight or prolonged bed rest leads to rapid losses of muscle mass and strength, recapitulating features of ageing. Accumulating evidence indicates that this exercise-induced muscle adaptation is driven by damage-evoked immune signaling that mobilizes bone marrow-derived progenitor cells to sites of tissue injury for regeneration. Cross-age transplantation studies further demonstrate that circulating bone marrow-derived cells (i.e., immune and progenitor cells) are key determinants of muscle regenerative capacity. Recent human muscle biopsy studies reveal that infiltrating immune and progenitor cells can fuse with damaged myofibers and contribute mitochondria during recovery. Within this damage-induced regeneration framework, ginsenosides, the bioactive steroidal constituents of *Panax* species, have emerged as potential modulators of immune activation, stem/progenitor cell mobilization, and cell-state regulation. However, randomized controlled trials using different ginseng extracts have yielded inconsistent outcomes in exercise adaptation, likely due to variability in ginsenoside composition across species, cultivation season, and processing. To date, rigorously controlled, double-blind trials using standardized ginsenoside remain scarce. Rg1 is the only compound supported by human biopsy evidence, associated with reproducible reductions in perceived exertion and senolytic effects following exercise-induced muscle damage. This review reports current evidence on ginsenosides, with a specific focus on Rg1, within an attrition-regeneration framework and proposes a testable mechanistic model to guide future human trials and translation.

## Highlights

• Exercise-induced muscle regeneration and strength gains are driven by immune signaling triggered by tissue damage.
• Bone marrow-derived cells fuse with injured myofibers, donating nuclei and mitochondria to support regeneration.
• Ginsenoside Rg1 modulates immunity, reduces perceived exertion, and enhances exercise-driven declines in cellular senescence.
• Standardizing ginsenoside composition is critical for developing consistent, effective ginseng-based nutraceuticals.

## Graphical abstract

Resistance exercise increases muscle strength and mass by inducing focal myofiber damage that triggers an inflammatory regenerative response. This immune response clears damaged components and mobilizes bone-marrow–derived progenitor cells, driving muscle rejuvenation and neuromuscular remodeling. Central neural command sets exercise intensity and thus shapes the extent of damage and regenerative strengthening. Pre-exercise ginsenoside Rg1 supplementation lowers perceived exertion, enabling a greater training stimulus and thereby enhancing bone-marrow cell mobilisation, supporting neural stem-cell activation, and amplifying exercise-associated reductions of stem-cell senescence in human skeletal muscle.

**Figure:**
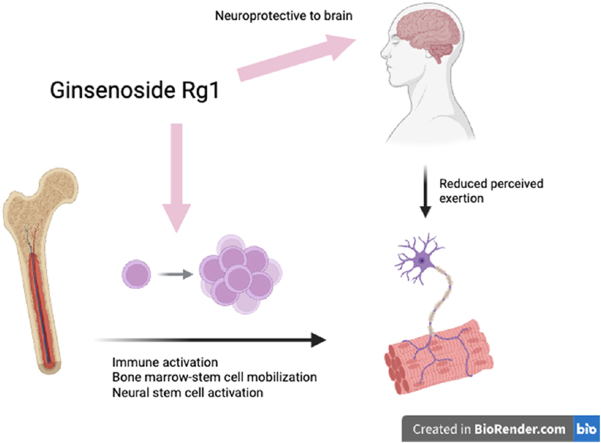
Resistance exercise increases muscle strength and mass by inducing focal myofiber damage that triggers an inflammatory regenerative response. This immune response clears damaged components and mobilises bone-marrow–derived progenitor cells, driving muscle rejuvenation and neuromuscular remodeling. Central neural command sets exercise intensity and thus shapes the extent of damage and regenerative strengthening. Pre-exercise ginsenoside Rg1 supplementation lowers perceived exertion, enabling a greater training stimulus and thereby enhancing bone-marrow cell mobilisation, supporting neural stem-cell activation, and amplifying exercise-associated reductions of stem-cell senescence in human skeletal muscle.

## 1 Introduction

Skeletal muscle adaptation is often framed as a metabolic consequence of repeated contraction and signaling. However, a growing body of evidence indicates that durable remodeling of human skeletal muscle fundamentally depends on a damage-evoked, immune-coordinated repair process that determines the efficiency and quality of myofiber regeneration under both normal weight loading conditions and exercise-induced stress.^1^, ^2^, ^3^ Mechanical loading generates focal structural disruption in susceptible myofibers, which in turn activates inflammatory signaling cascades that recruit immune and progenitor populations to restore tissue integrity and functional capacity. Adaptation should therefore be viewed not solely as a metabolic response to work performed, but as an organized inter-cellular response (from bone to muscle) to myofiber disruption.

In this review, we synthesize evidence into an attrition-regeneration framework in which mechanical loading induces focal damage, triggers immune activation, mobilizes bone-marrow-derived cells, and drives targeted renewal of weakened or injured myofibers. Recent human muscle biopsy studies extend prevailing models by demonstrating that infiltrating bone marrow-derived cells can contribute cytoplasmic material, including mitochondria and nuclei, to damaged myofibers during early recovery.^4^ indicating that muscle adaptation involves coordinated multicellular interactions rather than intrinsic myofiber remodeling alone. The following sections trace the sequence from focal disruption to targeted repair and then consider where ginsenosides, particularly Rg1, may plausibly influence these processes.

Within this context, we evaluate ginsenosides, the bioactive steroidal constituents of *Panax* species, as potential modulators of damage-induced myofiber regeneration. Rather than acting as direct anabolic agents, ginsenosides, particularly Rg1, are considered here as immune-regulatory adjuncts that may lower perceived exertion, permit greater mechanical loading, and thereby amplify engagement of regenerative and senescence-clearing pathways during exercise recovery. Translation of these effects has been limited by substantial heterogeneity in ginsenoside composition of ginseng extract across human trials, underscoring the need for standardized formulations and mechanistically grounded study designs.

Accordingly, this review does not attempt to summarize all pharmacological actions of ginseng preparations. Instead, we focus specifically on the role of bone marrow cells (immune cells and progenitor cells) governing exercise-induced skeletal muscle regeneration and examine whether ginsenoside Rg1, the only ginseng-based chemical component evaluated using controlled human biopsy studies, can modulate these processes. The aim is therefore translational: to integrate human physiological evidence with mechanistic biology and generate testable hypotheses regarding how a ginsenoside may interact with exercise-induced tissue repair.

## 2 Review methodology

**Fig. 1:**
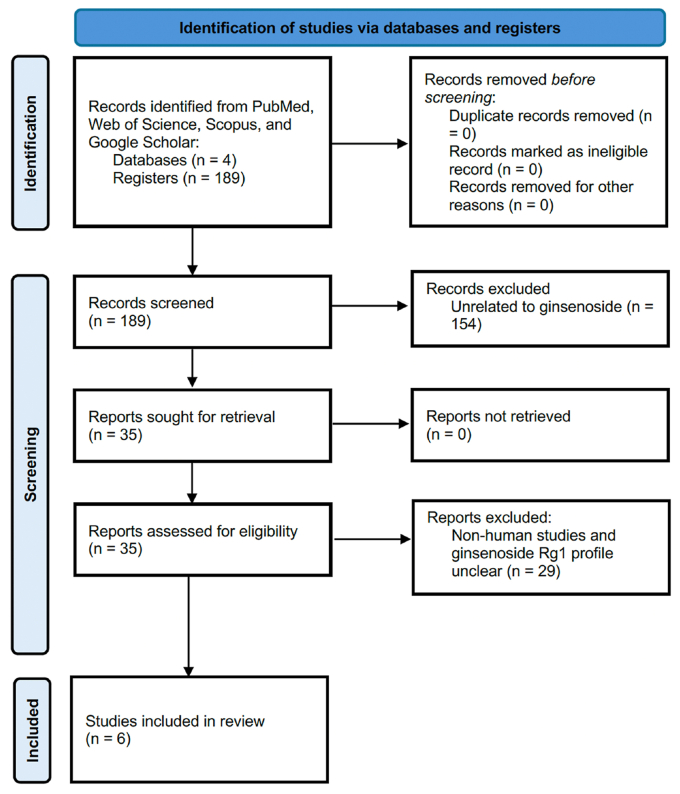
Consort flow chart of RCT using ginsenosides.

This narrative review followed structured reporting principles informed by PRISMA guidance for scoping reviews. Literature was searched in PubMed, Web of Science, Google Scholar, and Scopus from database inception to April 2026. The Search terms included (“ginsenoside Rg1″) AND (“skeletal muscle" OR "exercise" OR "regeneration" OR "satellite cell" OR "senescence" OR "inflammation"). Inclusion criteria were 1) Human studies evaluating exercise or skeletal muscle outcomes; 2) Randomized or controlled clinical trials of isolated or standardized ginsenoside; 3) Animal and *in vitro* studies were used for examining mechanistic insight on immune regulation, stem cell mobilization, or mitochondrial transfer relevant to muscle repair. Exclusion criteria were 1) Herbal mixtures without quantified ginsenoside composition; 2) Studies lacking identifiable skeletal muscle outcomes; 3) Pharmacokinetic or unrelated organ-system studies. Evidence weighting prioritised: 1) Human muscle biopsy trials were considered primary evidence; 2) Animal and cell culture studies were used only to support biological plausibility and were explicitly interpreted as hypothesis-generating rather than confirmatory; 3) When findings differed, interpretation considered compound standardisation, exercise model, and outcome relevance to regeneration biology. A flow diagram of study selection is provided in Fig. 1.

## 3 Muscle damage as the initiating stimulus

Human skeletal muscle consists of elongated, multinucleated myofibers approximately 50 μm in diameter that routinely bear mechanical loading. Under Earth's gravity (∼9.8 m s^−2^), body mass imposes persistent mechanical strain on these structures. Despite this, histological evidence indicates that only a small proportion (∼1%) of muscle cross-sectional area shows nucleated cell aggregation within collapsed or shrinking myofibers in healthy young adults.^5^ reflecting highly efficient regenerative mechanisms that preserve muscle integrity and contractile function.

When myofibers are weakened or damaged, they release damage-associated molecular signals, including cytokines and chemokines.^6^ that recruit immune cells to initiate debris clearance and progenitor cells for coordinated tissue renewal.^7^ This inflammatory response can extend beyond focal myofiber repair to support restoration of the local microenvironment, including neural and vascular elements in muscle tissues. Effective regeneration depends on the coordinated mobilization of immune cells for phagocytosis and progenitor cells for tissue renewal, many of which originate from bone-marrow stem-cell pools.^2^^,^^8^

Collectively, damage-induced inflammation represents a central physiological mechanism by which skeletal muscle undergoes dynamic rejuvenation *in vivo*, producing rapid reductions in senescent cell burden and with repeated exposure, gains in muscle mass and function.^1^^,^^3^

## 4 Muscle regeneration by bone-marrow-derived cells

Circulating blood cells of bone-marrow origin are key determinants of muscle regenerative capacity.^9^ Classic cross-age muscle transplantation experiments show that aged muscles grafted into young hosts regenerate comparably to young grafts in young hosts, whereas young muscles transplanted into aged hosts acquire impaired regeneration phenotype.^10^ Exposure of aged muscle to young circulating blood enhances recovery after injury by restoring regenerative signaling in aged satellite cells.^9^

These findings demonstrate that muscle regeneration is regulated not solely by intrinsic myofiber properties or resident stem cells, but also by circulating bone-marrow-derived cells and systemic factors that shape the regenerative environment.

## 5 Nuclear donation in skeletal muscle fiber regeneration

Skeletal muscle fibers are large, post-mitotic syncytia that increase myonuclear number during growth and repair through fusion with surrounding mononuclear myogenic cells. These cells were originally identified as satellite cells based on their position beneath the basal lamina around muscle fibers.^11^ Importantly, the satellite cell pool is heterogeneous and bone-marrow-derived lineages have been reported to contribute to regenerating myofibers via fusion in injury models.

Transplantation studies using a single haematopoietic stem cell have reported contributions to both haematopoietic reconstitution and skeletal muscle regeneration, consistent with fusion-mediated nuclear donation under inflammatory conditions.^12^^,^^13^ These *in vivo* observations challenge the classical Haematopoietic Stem Cell (HSC) – Mesenchymal Stem Cell (MSC) dichotomy derived largely from *in vitro* studies.^14^ Although bone-marrow-derived cell fusion into myofibers is less frequent under basal conditions, muscle injury markedly enhances this process.^15^ CD34^+^ haematopoietic progenitors contribute to both vascular endothelial regeneration and myogenesis following exercise-induced injury, while adipose-derived stem cells facilitate structure repair via lipid mobilization, i.e., lipolysis to release fatty acid to reconstitute cell membrane during cell regeneration.^16^ Muscle regeneration therefore appears to be a multicellular process with bone-marrow contributions.

## 6 Mitochondrial transfer to damaged human myofibers

**Fig. 2:**
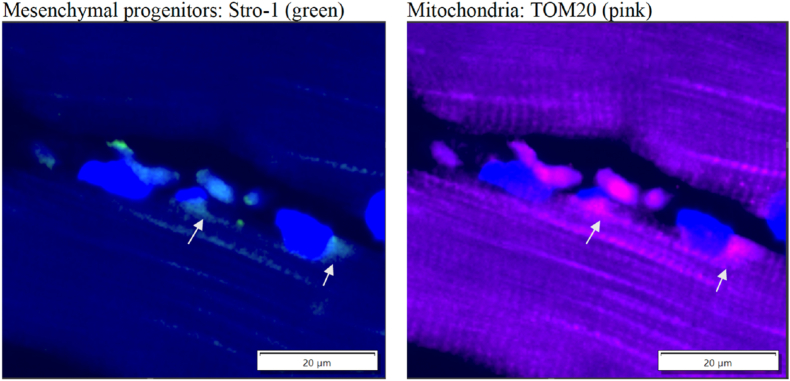
**Mitochondrial donation from** Stro-1^+^**progenitor cells to injured myofibers in exercised human skeletal muscle.** Infiltrating progenitor cells (Stro-1^+^ cells, green) display sharply higher mitochondrial density than engaged myofibers at damage sites. Mitochondria are labeled with anti-TOM20 (pink), and nuclei with DAPI (blue). Rg1 increases mitochondrial-rich Stro-1^+^ cell abundance in aging human skeletal muscle following resistance exercise.^17^ Arrows indicate docking and fusion of mitochondria-rich bone marrow-derived progenitor cells with muscle fibers.

Human muscle biopsy studies show that bone marrow–derived cells, characterized by a substantially higher mitochondrial content, infiltrate damaged myofibers and contribute mitochondria along with other cytoplasmic components.^4^ These mitochondria-rich cells (i.e. Stro-1^+^ progenitors) preferentially localize to focal damage sites.^17^ with enrichment of mitochondria extending into the adjacent myofiber cytoplasm (Fig. 2), in which injured myofibers acquire more mitochondria by cell docking and fusion. Infiltrating cells include myeloid lineages, including neutrophils and other immune cells as well as progenitor populations described elsewhere in this review.^2^^,^^4^ Intercellular mitochondrial transfer from donor cells of bone marrow origin is increasingly recognized as a conserved stress-response mechanism across tissues, including lung epithelium, nervous tissue, and myocardium.^18^^,^^19^

Erythroid cells remain underexplored as potential mitochondrial donors. Although mature erythrocytes lack organelles, reticulocytes contain mitochondria during early differentiation and are produced in large numbers daily. Mitochondrial clearance begins during the erythroblast-to-reticulocyte transition via programmed mitophagy.^20^ Whether mitochondria from erythroid lineage cells are exclusively degraded as a waste or can be supplied by damaged muscle tissues under inflammatory conditions remains unknown.

## 7 Lessons from spaceflight: muscle size and function requires damage-repair cycling

Traditional models attribute muscle adaptation in muscle mass and strength gains primarily to intermittent contraction and acute metabolic perturbation within myofibers. Spaceflight provides a natural experiment that challenges this view. Despite maintained neural activation and structured in-flight exercise, astronauts experience profound muscle atrophy and strength loss after the removal of mechanical stress on muscle fibers.^21^^,^^22^ Across space missions, muscle mass can fall by ∼20% within two weeks and losses in explosive force of up to ∼50% have been reported after long-duration flight.^23^ Muscle power and rate of force development also show disproportionate sensitivity to unloading and disuse,^24^ while biopsies indicate fiber atrophy together with downregulation of mitochondrial and oxidative machinery despite exercise countermeasures.^25^ Bed-rest studies reproduce these findings under terrestrial unloading in absence of cosmic radiation exposure as a potential confounding factor.^26^ Therefore, these observations suggest that acute metabolic signaling during intermittent contraction alone may be insufficient. The mechanical loading that normally engages damage-evoked regeneration processes by inflammation (immune cell infiltration into muscle tissues) is necessary for sustained muscle size and function.^8^

## 8 Inflammation is required for exercise-induced muscle regeneration

Resistance exercise often induces transient muscle fiber damage and inflammation, which strongly stimulate muscle hypertrophy.^2^^,^^27^ Inflammatory signaling mobilizes immune and progenitor cells via cytokine- and chemokine-mediated pathways.^2^^,^^28^ Disrupting macrophage recruitment or cyclooxygenase signaling can impede hypertrophy despite mechanical loading in injured animal models.^8^ Likewise, high-dose anti-inflammatory drug use in humans can attenuate training-induced gains in muscle mass.^29^^,^^30^ Findings vary with dose, population and training design. These studies therefore support the view that the exercise-induced muscle adaptation is driven by inflammation. Furthermore, high-intensity exercise (i.e. acute: >80% VO_2max_ in aerobic exercise or >80% 1RM in resistance exercise) induces senescent cell clearance and stem-cell rejuvenation to make muscle tissue younger.^3^^,^^5^^,^^31^ effects absent with energy-matched low-intensity exercise that produces minimal tissue disruption.^5^ Muscle stem cell activation after exercise is typically evident during the first 24-72 h of recovery, alongside transient mobilisation of circulating CD34^+^ cells.^32^ Overall, these findings establish beneficial muscle adaptation in size and function as an injury-driven, immune-dependent process rather than a simple consequence of energy expenditure.

## 9 Regulation of muscle stem-cell activation states

Stem cells involved in tissue regeneration exist in both quiescent and activated states. Quiescent stem cells exhibit low mitochondrial activity and rely primarily on glycolytic metabolism, preserving long-term regenerative capacity over extended periods. Redox signaling (i.e. ROS) induces their activation enabling repair of damaged myofibers by promoting mitochondrial biogenesis of the progenitor cells. Thus, effective muscle regeneration requires coordinated transitions from quiescent to activated stem cell states.^33^

Both insufficient activation and chronic activation impair regeneration. Inadequate stem cell activation results in ineffective repair due to an insufficient pool of activated stem cells.^33^ whereas persistent activation leads to stem cell exhaustion and subsequent fibrosis. Thus, effective muscle adaptation depends on tightly regulated state transitions (i.e., appropriate temporal cycling) rather than maximal activation. Metabolic cues, redox signaling, and inflammatory mediators collectively regulate this balance. The ginsenoside Rg1 from ginseng enhances abundance of activated stem cells, characterized by a high mitochondrial content, in human skeletal muscle following exercise.^17^

## 10 Reactive oxygen species (ROS) as a regulatory switch

ROS act as critical signaling molecules regulating stem-cell state transitions. During muscle injury, ROS generated by activated phagocytes can contribute to the local cues that promote stem-cell activation.^34^ High-dose antioxidant supplementation attenuates exercise-induced hypertrophy and strength gains in humans.^35^ Lowering post-exercise neutrophil response and ROS levels with a thiol-based antioxidant suppresses anabolic signaling pathways in exercised human skeletal muscle, including Akt/mTOR phosphorylation, p38 MAPK activation, and the myogenic response (MyoD induction).^36^ These studies implicate the necessity of ROS signaling for muscle stem cell activation.

Nutrient intake acutely elevates glucose and amino acid availability (increasing advanced glycation end products), leading to a transient increase in circulating ROS for approximately 2 h.^37^^,^^38^ whereas insulin-mediated glucose lowering reduces ROS production.^39^ This acute phase postprandial ROS pulse facilitates immune activation and the clearance of damaged or senescent cells as nutrients are available.^40^ However, persistent accumulation of advanced glycation end products under hyperglycemic conditions can result in bone marrow cell exhaustion to impair regenerative capacity. In contrast, excessive nutrient restriction (i.e. long-term fasting or metformin) can blunt exercise-induced muscle repair due to lack of activated stem cells.^33^^,^^41^ Both extremes reflect a loss of homeostatic balance in the ratio between activated and quiescent stem-cell states. Together, the timing of meals relative to ginsenoside supplementation may potentially impact key outcomes in muscle mass and strength during exercise training.

## 11 Rb1 and Rg1: Major ginsenosides with opposing actions

Rb1 (15–30%) and Rg1 (5–15%) are among the most abundant ginsenosides present in *Panax* preparations.^42^ and exhibit contrasting physiological effects in several biological actions.^43^^,^^44^ Sengupta et al. (2004) demonstrated opposing actions of angiogenesis: ginsenoside Rg1 stimulated endothelial cell proliferation, migration, and tube formation, consistent with a pro-angiogenic phenotype mediated through nitric-oxide signaling and activation of PI3K/Akt pathways.^43^ In contrast, Rb1 inhibited angiogenesis by suppressing endothelial proliferation and vessel formation. A later physiological study reported a comparable divergence in metabolic regulation, in which Rg1 decreased postprandial glycemia after an oral glucose challenge, whereas Rb1 increased postprandial glycemia under the same condition.^44^

These findings collectively indicate that Rg1 predominantly supports processes associated with tissue repair and activation (angiogenesis, cell migration, and metabolic mobilization), whereas Rb1 favors metabolic homeostasis and growth restraint. Ginsenoside profiles of Rg1 and Rb1 are changing with geographic origin, cultivation conditions, harvest season, and processing methods, leading to inconsistent metabolic effects across studies.^45^, ^46^, ^47^.

It is noteworthy that most human trials using ginseng extracts have neither controlled nor quantified ginsenoside composition, a limitation that likely contributes to heterogeneous findings. A meta-analysis of 12 randomized controlled trials using ginseng without controlled ginsenoside profile (n = 630) reported variable effects on physical performance despite relatively consistent reductions in subjective fatigue.^47^ These observations underscore the need for standardized ginsenoside formulations or investigations using isolated compounds. Notably, no randomized human trials of Rb1 have been reported, whereas Rg1 has been examined in controlled human studies.

## 12 Immunomodulatory actions of ginsenoside Rg1

The immunomodulatory properties of ginsenosides are well established in animal models.^48^^,^^49^ Rg1 regulates both innate immune and progenitor cell activity. In macrophages, Rg1 suppresses excessive pro-inflammatory cytokine production (e.g., TNF-α, IL-6, IL-1β, and iNOS) in animal models.^50^ while promoting a shift toward a resolving M2-like phenotype. In humans, this is reflected by increased IL-10 production and reduced TNF-α expression in skeletal muscle following exercise.^51^ This regulatory pattern is consistent with more efficient debris clearance and a shortened inflammatory phase. Rg1 also enhances haematopoietic progenitor cell mobilization via activation of the stromal cell–derived factor-1 (SDF-1)/C-X-C chemokine receptor type 4 (CXCR4) chemotactic axis, which governs bone marrow–derived cell homing to injured tissues.^52^ In addition, Rg1 increases the migratory and angiogenic capacity of endothelial progenitor cells, suggesting a role in restoring microvascular integrity after injury.

Collectively, evidence from previous *in vitro* and animal studies suggests that Rg1 may facilitate the resolution phase of inflammation following exercise, a stage essential for effective regeneration but often impaired with ageing and disuse. Importantly, these effects appear to be contingent on the presence of tissue damage; in the absence of injury-related signals, Rg1 alone may not induce muscle hypertrophy without exercise training.^53^

## 13 Human muscle biopsy evidence for Rg1

Randomised controlled trials (RCT) incorporating standardized ginsenoside profiles and skeletal muscle biopsies provide the most rigorous human evidence, summarised in Table 1. Rg1 supplementation does not alter maximal oxygen uptake but increases endurance performance during high-intensity exercise (assessed by time to exhaustion at 80% VO_2max_).^51^ likely associated with lower perceived exertion during exercise.^31^ Importantly, pre-exercise Rg1 supplementation has been associated with enhanced exercise-induced senolytic action, with reductions in senescence-associated β-galactosidase (SA-β-gal)-positive cells observed only when combined with mechanical cycling at moderate intensity.^54^ These effects are associated with mobilization of activated stem cells in exercised muscle. In women aged 60-73, pre-exercise Rg1 supplementation significantly increases the abundance of activated stem cells (mitochondrial-rich fraction) in exercised skeletal muscle.^17^ These findings suggest a role for Rg1 as an immune-regulatory adjunct to exercise for a hypertrophic agent. It is worth noting that supraphysiological ginsenoside doses in the hundreds-of-milligrams range have been associated with immune- or hypersensitivity-related adverse events, underscoring the importance of dose standardization in application.^55^

**Table 1:** Human RCTs evaluating ginsenoside effect on exercise-induced muscle adaptation.

Citation	Design	Dose	Key Outcome
Hou et al.^51^	Mean age 21 y; 2-arm crossover RCT; 60-min aerobic cycling at 70% VO_2_max	Ginsenoside Rg1 (10 mg (5 mg night before + 5 mg 1 h pre-exercise)	Muscle TNF-α mRNA: ↓ Muscle IL-10 mRNA: ↑ Muscle IL-6 mRNA: no difference VO_2max_: no effect Time to exhaustion: ↑ (∼20%) Muscle glycogen depletion: ↑
Lee et al.^55^	Mean age 42 y; 3-arm RCT; 12-week resistance training	Ginsenoside-enriched extract (Rd, Rg3, Rg5, Rk1); low dose 100 mg/d (n = 39), high dose 500 mg/d (n = 39), placebo 500 mg/d (n = 39)	Anaerobic threshold: no effect VO_2max_: no effect Muscle strength: ↑ at both doses Immune- or inflammation-related adverse events: 13% (e.g., allergic reaction, mild pruritus, upper respiratory infection, knee pain)
Wu et al.^54^	Mean age 21 y; 2-arm crossover RCT; acute 60-min aerobic cycling at 70% VO_2_max	Ginsenoside Rg1, 5 mg (1 h pre-exercise)	Muscle senescence (SA-β-gal): ↓ Apoptotic DNA fragmentation: +30% (NS) Leukocyte infiltration: +30% (NS) Resolution of DNA fragmentation: 35% ↑ Resolution of leukocyte infiltration: 30% ↑ Recovery from muscle fibrosis: ↑
Wu et al.^56^	Mean age 21–23 y; 2-arm crossover RCT; acute 60-min aerobic cycling at 70% VO_2_max	Ginsenoside Rg1, 5 mg (1 h pre-exercise)	Pax7 muscle stem cell depletion, glutathione depletion, and TNF-α mRNA increases: blocked Post-exercise myogenesis: ↑ Resolution of inflammation in muscle infiltrating cells: ↑
Lee et al.^31^	Mean age 22 y; 2-arm crossover RCT; acute resistance exercise (6 × 8 reps at 70% 1-RM)	Ginsenoside Rg1 (Senactiv®), 5 mg (1 h pre-exercise)	Perceived exertion: ↓ Muscle senescence marker p16^INK4a^ mRNA: ↓
Nicholls et al.^17^	Age 60-73 y; 2-arm crossover RCT; acute resistance exercise (4 × 8 reps at 70% 1-RM)	Ginsenoside Rg1 (Senactiv®), 10 mg (1 h pre-exercise)	Stro-1^+^ progenitor cells: ↑ Nestin^+^ neural stem cells: ↑ Mitochondria: ↑ Estrogen depletion during exercise recovery: blocked Circulating progesterone: ↑

Inclusion criteria: human trials; exclusion criteria: non-human or in vitro studies; upward and downward arrows: enhance and suppress; NS: no significance.

## 14 Neural and motor unit remodeling by Rg1

Skeletal muscle adaptation is not limited to myofiber regeneration but also requires restoration of neuromuscular junctions. A substantial body of cell culture and animal research indicates that ginsenoside Rg1 robustly stimulates neurogenesis. Following injury, denervated fibers must be reinnervated to recover force production. Emerging evidence suggests that Rg1 promotes neural progenitor proliferation and neurite outgrowth via the PI3K/Akt and ERK signaling pathways.^57^ Rg1 increases neurite length and reduces apoptosis, indicating both neuroprotective and neurotrophic effects mediated specifically through activation of Akt and ERK1/2; blockade of either pathway abolishes neurite outgrowth and protection against amyloid-β–induced cell death.^57^ Rg1 also activates neural stem cells, as shown by enhanced neuronal differentiation *in vitro*.^58^ and increased proliferative activity *in vivo*, evidenced by ^3^H-thymidine incorporation and a greater number of proliferating progenitor neurospheres in adult mice.^59^ Furthermore, Rg1 promotes transdifferentiation toward neural stem-like phenotypes in adipose-derived stem cells.^60^ and human umbilical cord–derived mesenchymal stem cells under defined conditions.^61^

In humans aged >60 years, Rg1 appears to increase neural stem cell abundance, differentiation, and mobilization to sites of injury in exercise-challenged skeletal muscle.^17^ consistent with animal findings.^62^ Taken together, these observations provide a plausible mechanistic basis for the reduced perceived exertion reported in human Rg1 trials.^17^^,^^31^ Improved neuromuscular transmission may reduce motor unit recruitment requirements for a given force output.

Beyond lineage commitment, Rg1 enhances stem cell proliferation and migratory capacity 62, 63, 64, effects likely mediated via activation of the SDF-1/CXCR4 signaling axis.^6^ However, it remains unclear whether sustained Rg1-induced activation predisposes stem cell populations to exhaustion *in vivo*, a concern that may be particularly relevant in older adults.

Mitochondrial enrichment is a hallmark of stem cell activation from quiescence. In mice, Rg1 preserves mitochondrial cristae integrity and contractile function in cardiac muscle following endotoxin injury.^65^ This protective effect is consistent with enhanced mitochondrial contribution to human skeletal muscle from bone marrow–derived cells.^17^

## 15 Conclusion

Skeletal muscle adaptation in mass and strength is driven in large part by damage-induced, immune-coordinated regeneration. Bone marrow-derived cells (immune and progenitor populations) contribute to the renewal of injured myofibers through mitochondrial transfer during early recovery and nuclear accretion via cell fusion during repair. Human evidence supporting mitochondrial redistribution/transfer and bone marrow–derived cell fusion during and after exercise has emerged from muscle biopsy studies and is strengthen by transplantation-based lineage-tracing data from preclinical models.^66^ Accordingly, the framework presented here integrates these data into a coherent, testable model that identifies where ginsenoside Rg1 may plausibly modulate damage-to-repair biology in conjunction with mechanical loading.

Collectively, the evidence is consistent with an attrition-regeneration framework in which focal disruption recruits immune and progenitor populations to coordinate targeted repair, thereby shaping longer-term gains in muscle mass and function. Within this framework, ginsenosides, particularly Rg1, are best viewed as immune modulators that may enhance regenerative efficiency when combined with mechanical loading, partly by lowering perceived exertion and modulating senescence-related processes. Nutraceutical translation of ginseng products is currently constrained by incomplete standardization and inconsistent reporting of ginsenoside composition across trials. Rigorous, well-controlled human studies, including those examining other ginsenoside species, are needed to define their nutraceutical or pharmacological potential for preserving muscle health during ageing, disuse, and physical inactivity.

## Funding acknowledgements

This review article is sponsored by The 10.13039/501100010410Education University of Hong Kong.

## Declaration of competing interest

The author declares prior research involvement in the development of Senactiv®, supported by research funding from Nuliv Science USA. This funding was not related to the preparation of the present review. No commercial entity had any role in the writing, interpretation, or conclusions of this manuscript.
